# RDD-HCD Provides Variable Fragmentation Routes Dictated by Radical Stability

**DOI:** 10.1021/jasms.2c00326

**Published:** 2023-02-14

**Authors:** Jacob W. Silzel, Ryan R. Julian

**Affiliations:** Department of Chemistry, University of California, Riverside, California 92521, United States

**Keywords:** fragmentation, photodissociation, radical-directed dissociation, higher-energy collisional dissociation, collision-induced dissociation

## Abstract

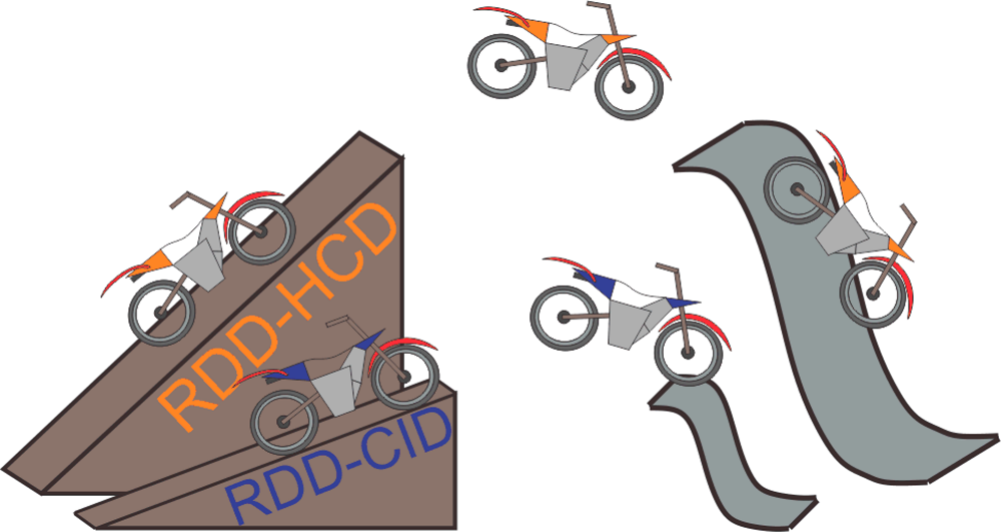

Radical-directed dissociation (RDD) is a fragmentation technique in which a radical created by selective 213/266 nm photodissociation of a carbon–iodine bond is reisolated and collisionally activated. In previous RDD experiments, collisional activation was effected by ion-trap collision-induced dissociation (CID). Higher-energy collisional dissociation (HCD) differs from CID both in terms of how ions are excited and in the number, type, or abundance of fragments that are observed. In this paper, we explore the use of HCD for activation in RDD experiments. While RDD-CID favors fragments produced from radical-directed pathways such as a/z-ions and side chain losses regardless of the activation energy employed, RDD-HCD spectra vary considerably as a function of activation energy, with lower energies favoring RDD while higher energies favor products resulting from cleavage directed by mobile protons (b/y-ions). RDD-HCD therefore affords more tunable fragmentation based on the HCD energy provided. Importantly, the abundance of radical products decreases as a function of increasing HCD energy, confirming that RDD generally proceeds via lower-energy barriers relative to mobile-proton-driven dissociation. The dominance of b/y-ions at higher energies for RDD-HCD can therefore be explained by the higher survivability of fragments not containing the radical after the initial or subsequent dissociation events. Furthermore, these results confirm previous suspicions that HCD spectra differ from CID spectra due to multiple dissociation events.

## Introduction

Radical-directed dissociation (RDD) is a tandem mass spectrometry technique in which a radical is created site-specifically on a biomolecule and is subsequently activated by collisions to induce fragmentation. Prior to dissociation, the radical typically migrates to nearby sites by hydrogen atom abstraction, which lends a high degree of structural sensitivity to the method.^1^ Migration sites are determined by a combination of structural constraints and the relative bond-dissociation energies of the initial and final sites.^2^ Radicals can be created by addition of a chromophore such as 4-iodobenzoic acid, followed by highly specific photodissociation of the carbon–iodine bond or by addition of a functional group labile to collisional activation, such as Tempo.^3^ Radicals can then (in either case) be reisolated and subjected to additional collisional activation in an MS^3^ step. Differences in RDD fragmentation have allowed disambiguation of many classes of similar molecules, including lipid isomers varying only by the position of double bonds in the fatty acid chains.^4−8^ RDD can also distinguish glycan oligomers differing in composition, configuration, and connectivity.^9,10^ In particular, RDD is noted for sensitivity to stereochemistry, enabling distinction of glycosphingolipid epimers, which only varied by the orientation of a single OH group in either the axial or equatorial position.^11^ In similar applications, RDD has been used to distinguish isomeric peptides where a single side chain was inverted from the l to d configuration.^1^

RDD tends to produce different fragment ion types than those observed with traditional CID experiments on protonated analytes. For peptides, CID spectra are dominated by b/y fragments, while RDD generates primarily a/x and c/z backbone fragments in addition to partial and complete side chain losses.^2^ It is interesting to consider why RDD fragmentation is different from traditional CID, given that both methods involve the use of collisional activation. One hint can be derived from a very small minority of peptides that behave as antioxidants and are able to sequester radicals and yield RDD spectra dominated by b/y-ions.^12^ This suggests that in the absence of radical sequestration, RDD is favored over proton-initiated fragmentation, presumably by facilitating lower-energy dissociation thresholds.^2^ However, ion-trap CID is not well suited for varying energy deposition because ions are slowly heated by thousands of collisions, and products are cooled immediately after creation. In contrast, higher-energy collisional dissociation (HCD) potentially allows for more control over the amount of energy deposited into ions.

Although similar methods, HCD and CID differ significantly in the mechanism by which energy is delivered to ions. In ion-trap CID, ions are resonantly excited to cause energetic collisions with helium gas. During the entire excitation period, ions are accelerated from one collision to another, eventually acquiring enough energy in small increments to fragment. In HCD, a beam of precursor ions is accelerated into a collision gas cell, allowing for a smaller number of collisions but at higher energy per collision relative to CID. Additionally, the time scales of energy transfer are quite different, with CID activation occurring over milliseconds, while HCD takes only microseconds.^13^ In principle, these differences should allow precursors in HCD to be excited to higher energies than those attainable in ion-trap CID.^14^ In practice, HCD and CID spectra tend to contain many similar fragment ions, but they are not identical, and the use of higher HCD energies leads to larger differences between the observed spectra.^15^

These fundamental mechanistic differences lead to changes in the relative abundances of fragments made as well as some differences in types of fragments made, and the differences between HCD and CID have been the subject of significant discussion. For example, CID spectra of peptides tend to contain mostly a, b, or y fragments and neutral losses, while HCD spectra contain these same fragments as well as more internal fragments and immonium ions.^16^ In addition, higher HCD energies tend to reduce the abundance of larger fragments, presumably due to increased sequential fragmentation events that yield smaller fragments.^17^ With large molecules such as peptides or proteins, more degrees of freedom necessitate higher energies for dissociation, and fragments often have sufficient internal energy for further degradation.^18^ Since HCD provides more energy per collision than CID, the likelihood for sequential fragmentation is increased. Smaller fragment ions and singly charged fragment ions make up a larger percentage of peptide HCD spectra and can represent the dominant fragments in some cases.^19^ Large scale comparison of HCD and CID libraries revealed results consistent with previous observations.^20^

In this paper, we explore the differences between RDD-CID and RDD-HCD as well as track the changes in fragmentation observed as RDD-HCD energy is increased. Our results reveal that radical-based fragments are more favorably produced during RDD-CID and low-energy RDD-HCD, while b/y fragments and smaller ions dominate higher-energy RDD-HCD. In addition, a stark contrast between the abundance of radical species and nonradical species is observed as HCD energy is increased, providing insight into the relative energy thresholds of RDD versus mobile-proton-based dissociation.

## Experimental Section

### Materials

Organic solvents and reagents were purchased from Fisher Scientific, Sigma-Aldrich, or Acros Organics and used without further purification. FMOC-protected amino acids and Wang resins were purchased from Anaspec, Inc. or Chem-Impex International. β-endorphin (YGGFMTSEKS QTPLVTLFKN AIIKNAYKKG E) was purchased from AnaSpec Inc. (Cat # 24319), and RRLIEDNEYTARG was purchased from Enzo (Cat # BML-P307-0001). AKAKTDHGAEIVYK was synthesized according to a modified solid-phase peptide synthesis protocol.^21^

### Iodination and 4IB Modifications

β-endorphin was iodinated via reaction with NaI, chloramine-T, and sodium metabisulfite in a manner to prevent excess iodination. Briefly, NaI and chloramine T were combined in a 1:2 molar ratio prior to addition to β-endorphin. Following this, 1/3 mol equiv of NaI:chloramine-T was added to 20 μL of 1 mM β-endorphin in water and allowed to react for 3 min before addition of the next equivalent for a total of 1 mol equiv of NaI at 9 min. The reaction was then quenched with 4× molar equivalents of sodium metabisulfite. A 20 μL aliquot of 1 mM RRLIEDNEYTARG was iodinated by reaction of peptide, NaI, and chloramine T at a 1:1:2 molar ratio for 10 min. At 10 min, the reaction was quenched by the addition of 4× molar equivalents of sodium metabisulfite. AKAKTDHGAEIVYK was covalently modified with 4-iodobenzoic acid (4IB) via reaction with 4IB-N hydroxy succinimide (4IB-NHS). Briefly, 4IB-NHS was synthesized by reaction of 1:1:1 4IB:DCC:NHS (0.5 mmol ea.) in 15 mL of dioxane for 12 h under N_2_. After 12 h, the reaction precipitate was removed via filtration, and dioxane was gently evaporated with N_2_. Following this, covalent attachment of 4IB was achieved by reaction of 50 μg of AKAKTDHGAEIVYK in 25 μL of 100 mM borate buffer (pH 8.5) with 25 μL of 6.5 mM 4IB-NHS (10-fold molar excess) in dioxanes for 1 h. Iodo-RRLIEDNEYTARG and 4IB-AKAKTDHGAEIVYK were desalted on a MICHROM Bioresources peptide MicroTrap (P/N TR1/25109/02) directly following iodination to remove salts and reaction byproducts prior to MS analysis. Following iodination, iodo-β-endorphin was desalted on a MICHROM Bioresources protein MicroTrap (P/N TR1/25109/03).

### Radical-Directed Dissociation Experiments

**Figure 1:**
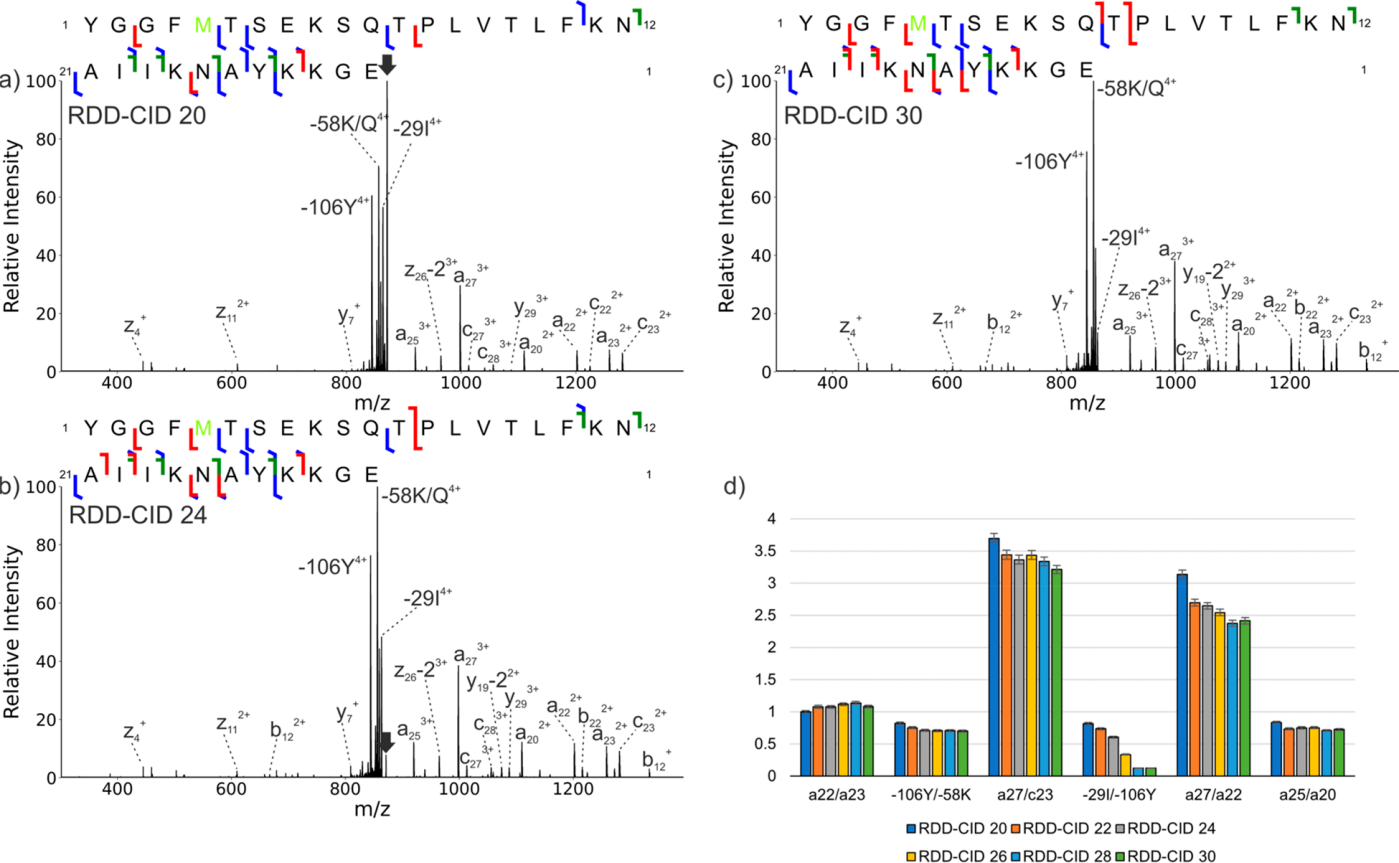
RDD-CID spectra and sequence ladders for β-endorphin at varying CID energies. (a–c) RDD-CID spectra for CID energies of 20, 24, and 30. (d) Ratios of intensities for selected fragment ion pairs a_22_/a_23_, −106Y/–58K, a_27_/c_23_, −29I/–106Y, a27/a22, and a25/a20. Error bars represent the standard deviation of the mean. The −29I/–106Y ratio exhibits the biggest change due to leakage of the excitation waveform fragmenting the −29I peak. In the sequence ladders, b/y fragments are shown in red, c/z fragments are in blue, and a fragments are in green.

All experiments were performed on a Thermo Orbitrap Fusion Lumos. Peptides were introduced into the instrument via direct infusion using either a HESI source or a modified nano flex source from Thermo Scientific. The nano flex source was modified with a platinum wire to allow use of tips pulled from borosilicate glass (Harvard Apparatus GC100T-10). Peptide sprayed with the HESI source was diluted to 1 μM in 50:50:0.1 H_2_O:ACN:FA (v/v/v), while peptide sprayed with the nano flex source was diluted to 1 μM in water with 0.1% FA. Peptides were isolated using the quadrupole prior to either 213 or 266 nm photodissociation in the low-pressure ion trap, after which the radical was reisolated for either CID or HCD fragmentation and analysis in the Orbitrap mass analyzer. For RDD-CID, a single normalized collision energy (referred to hereafter as CID energy) was selected, at which the precursor was no longer observed to be the base peak with the exception of the results in Figure 1 where details are given. For RDD-HCD, normalized collision energies (HCD energy) were varied to produce changes in fragmentation.

### Data Analysis

Following acquisition of data, deconvolution was performed in FreeStyle (v1.7) with Xtract with the analyzer type set to “OT”, isotope table set to “protein”, and the relative abundance threshold set to 1%. Fragment ions were assigned with a 0.01 Da tolerance. Following this, the fractional abundance of the deconvoluted data was calculated, where fractional abundance = intensity of fragment ion/sum of all fragment ion intensities. To aid in tracking the changes in fractional abundance as HCD energy is increased, % change was calculated using the first RDD-HCD energy as the initial point. % change was calculated as [(FRAB_n_ – FRAB_0_)/FRAB_0_ ] * 100. Sequence coverage plots were created using MASH (v2.2.0.33927). Side chain losses are common in RDD and are labeled by residue and approximate mass lost, i.e., −106Y indicates a 106 Da loss from the side chain of tyrosine.

## Results and Discussion

### RDD-CID vs RDD-HCD

To explore the effect of activation parameters on the relative abundances and fragment types observed in RDD-CID, experiments were performed on the 4+ charge state of iodo-β-endorphin at various CID energies (Figure 1a–c). The spectra are all quite similar, but a few minor differences can be noted. For example, the ions nearest to the precursor *m*/*z* are somewhat depleted at higher energies (i.e., −NH_3_/H_2_O and −29I). This is likely due to leakage of the excitation waveform into these nearby *m*/*z*’s. Other minor differences include a complementary pair of b_12_/y_19_-2 fragments, which are significantly more abundant at higher RDD-CID energies. To further quantify any changes in abundance as a function of CID energy, ratios of the relative intensities for a representative set of fragment pairs were calculated as shown in Figure 1d. Constant ratios indicate similar relative ion abundances at all CID energies, which was the trend observed for most ions. Overall, the results illustrate that increasing the activation energy in ion-trap CID does not appreciably change the resulting fragmentation. This can be explained by considering that after dissociation, ions will no longer be resonantly excited but will instead undergo cooling collisions. Since the input of energy is slow and takes place over many small steps, it is not possible to raise the precursor ion energy significantly over the RDD thresholds. Although the collision energy step can be changed somewhat by altering the activation *Q*, changing this parameter also had little effect on the results (Figure S1).

**Figure 2:**
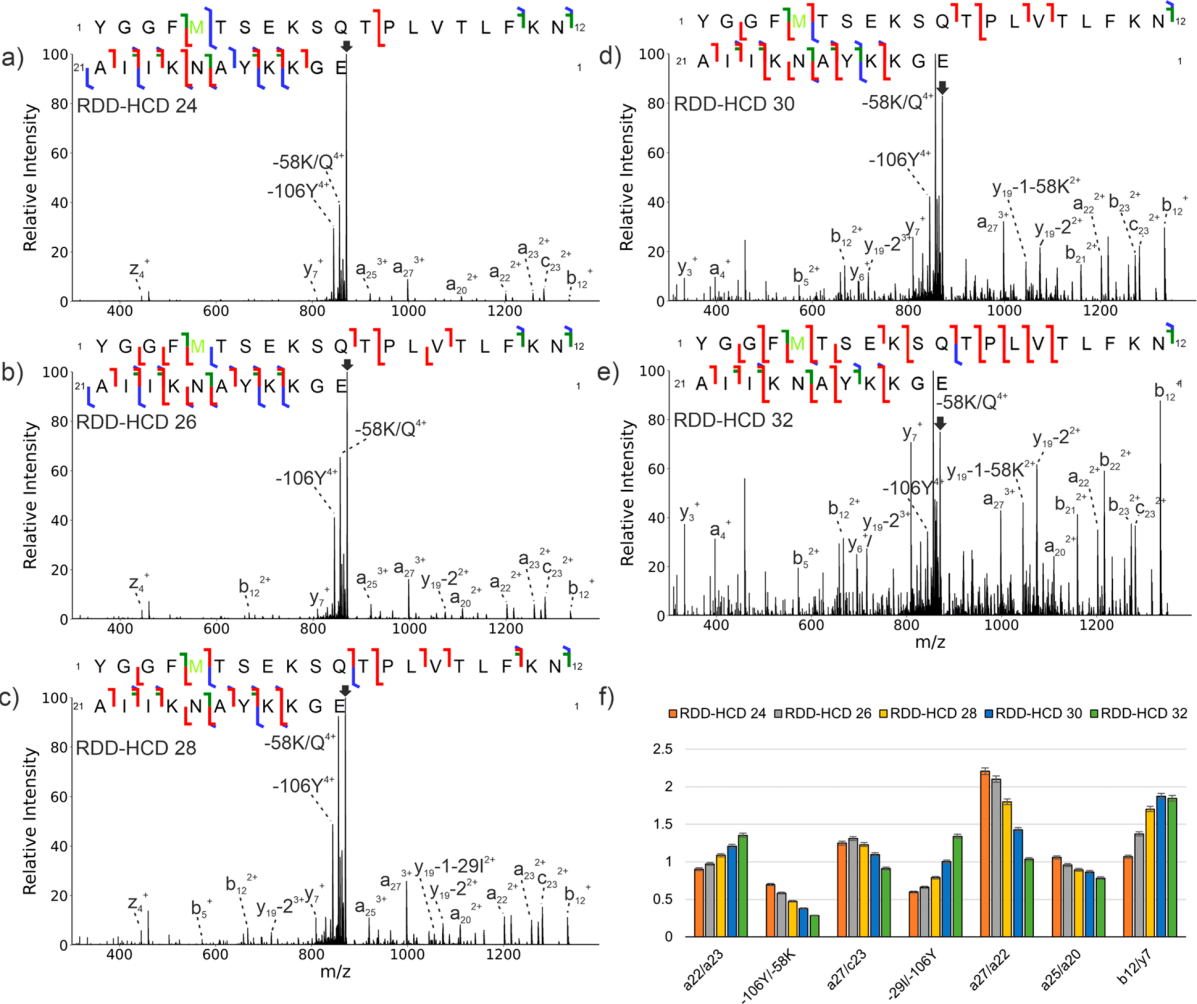
RDD-HCD spectra and sequence ladders for 4+ β-endorphin. (a) RDD-HCD with a normalized collision energy (NCE) of 24, (b) RDD-HCD, NCE 26, (c) RDD-HCD, NCE 28, (d) RDD-HCD, NCE 30, (e) RDD-HCD, NCE 32, and (f) ratio plots for a_22_/a_23_, −106Y/–58K, a_27_/c_23_, −29I/–106Y, a_27_/a_22_, a_25_/a_20_, b_12_/y_7_. Arrows indicate unfragmented precursor ion. In the sequence ladders, b/y fragments are shown in red, c/z fragments are in blue, and a fragments are in green.

Results for an analogous series of RDD-HCD experiments are shown in Figure 2. Even by casual observation, it is clear that the spectra in Figure 2 change significantly as energy is increased. At the lowest HCD energy, the number, type, and abundances of fragment ions are similar (though not identical) to those obtained by RDD-CID (compare Figures 1a and 2a). At higher energies, the number of fragment ions appears to increase, particularly in the lower *m*/*z* range, while side chain losses are reduced in fractional abundance, particularly for the −106Y side chain loss (Figure 2b–e). In contrast to RDD-CID, most of the fragment ion ratios tend to change as a function of energy in RDD-HCD as illustrated for several representative pairs in Figure 2f. Since precursor ions are largely accelerated prior to collisions in HCD, it is possible to access higher activation energies that facilitate alternative and/or sequential fragmentation pathways. Having noted some general trends, we now examine the effects of HCD energy on RDD product ions in greater detail.

### RDD-CID/HCD Neutral Loss Behavior with Increasing HCD Energy

**Figure 3:**
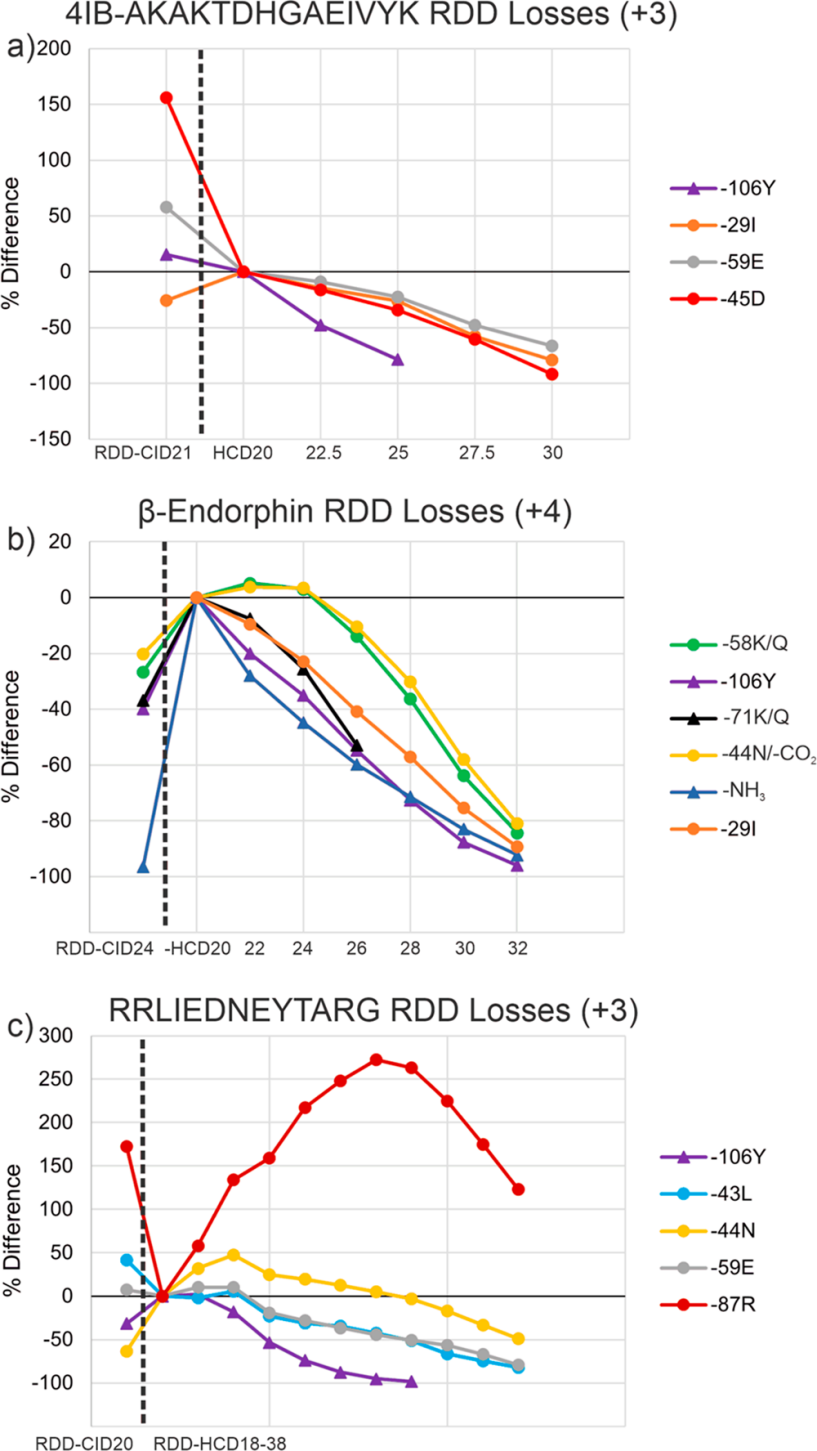
RDD-CID/HCD % change plots for the neutral loss fractional abundances for (a) the 3+ charge state of 4IB-AKAKTDHGAEIVYK, (b) the 4+ charge state of iodo-β-endorphin, and (c) the 3+ charge state of RRLIEDNEYTARG. The first point on the plots is the initial RDD-CID point, while the subsequent points are RDD-HCD fractional abundances with the HCD energy increased at regular intervals. The dashed line separates the RDD-CID point from the RDD-HCD points. Radical species are represented with triangles at each data point, and nonradical species are represented with circular points.

Previous work has shown that for most peptides in the tryptic size regime, RDD produces side chain losses from the original precursor ion by three general mechanisms (called type I, II, and III).^2^ To illustrate the behavior of these side chain losses as a function of activation energy, we plot the percent change in fractional abundance (FRAB) for several peptides in Figure 3. The percent change is referenced to the lowest RDD-HCD energy (the second data point), with RDD-CID represented by the first data point. Only one RDD-CID data point is shown, since RDD-CID spectra do not change appreciably with activation energy as shown previously in Figure 1. Experiments on the 3+ charge state of 4IB-AKAKTDHGAEIVYK reveal a decrease in most side chain losses upon transition from RDD-CID to RDD-HCD (Figure 3a). Subsequently increasing the HCD energy decreases the FRAB for all side chain losses, although the −106Y loss appears to be most labile. It is worth noting that the mechanism producing the −106Y loss leaves the radical species on the peptide while the −29I, −59E, and −45D losses leave behind even-electron peptides. These results suggest that radical species may more easily undergo subsequent fragmentation by HCD. Results for the 4+ charge state of β-endorphin are shown in Figure 3b. Interestingly for this peptide, all side chain losses are more abundant in low-energy HCD versus CID. At higher HCD energies, all of these products decrease in abundance, but again, those losses that produce radical peptides (−71K, −106Y, and −NH_3_) decrease more quickly. Very similar results are obtained for the 5+ charge state of β-endorphin (Figure S2), which may suggest that charge state does not play a significant role. The peptide fragments remaining behind after small molecule side chain losses are nearly the same size as the initial peptide precursors, which should increase the probability for energetic secondary collisions. Such large species will also have nearly the same number of low energy dissociation pathways still available as the original precursor ion; therefore, it is not surprising that further decay is observed at higher HCD energies. Similarly to the previous two examples, RDD-HCD experiments on RRLIEDNEYTARG result in decreases in FRAB for nearly all side chain loss products, with type III side chain −106Y exhibiting the quickest decrease in FRAB relative to the other side chain losses (Figure 3c). Interestingly, the −87R loss was found to increase substantially with HCD energy and only begins to decrease in FRAB at much higher HCD energies. This behavior is quite opposite to the trend observed for other side chain losses and may be related to the fact that −87R is not a neutral loss, since it contains a proton on the Arg side chain and leads to a reduction of charge state.

To further explore the stability of radical versus nonradical product ions, the peptides produced by side chain losses were reisolated and subjected to identical CID activation in MS^4^ experiments. −106Y and −71K exhibited a higher degree of fragmentation than the nonradical −58K loss (Figure S3). The FRAB of the intact −106Y, −71K, and −58K precursors was 0.18, 0.27, and 0.38, respectively. The higher residual precursor for the nonradical −58K loss is consistent with higher-energy dissociation barriers for this fragment.

### Stability of a-Ions

**Figure 4:**
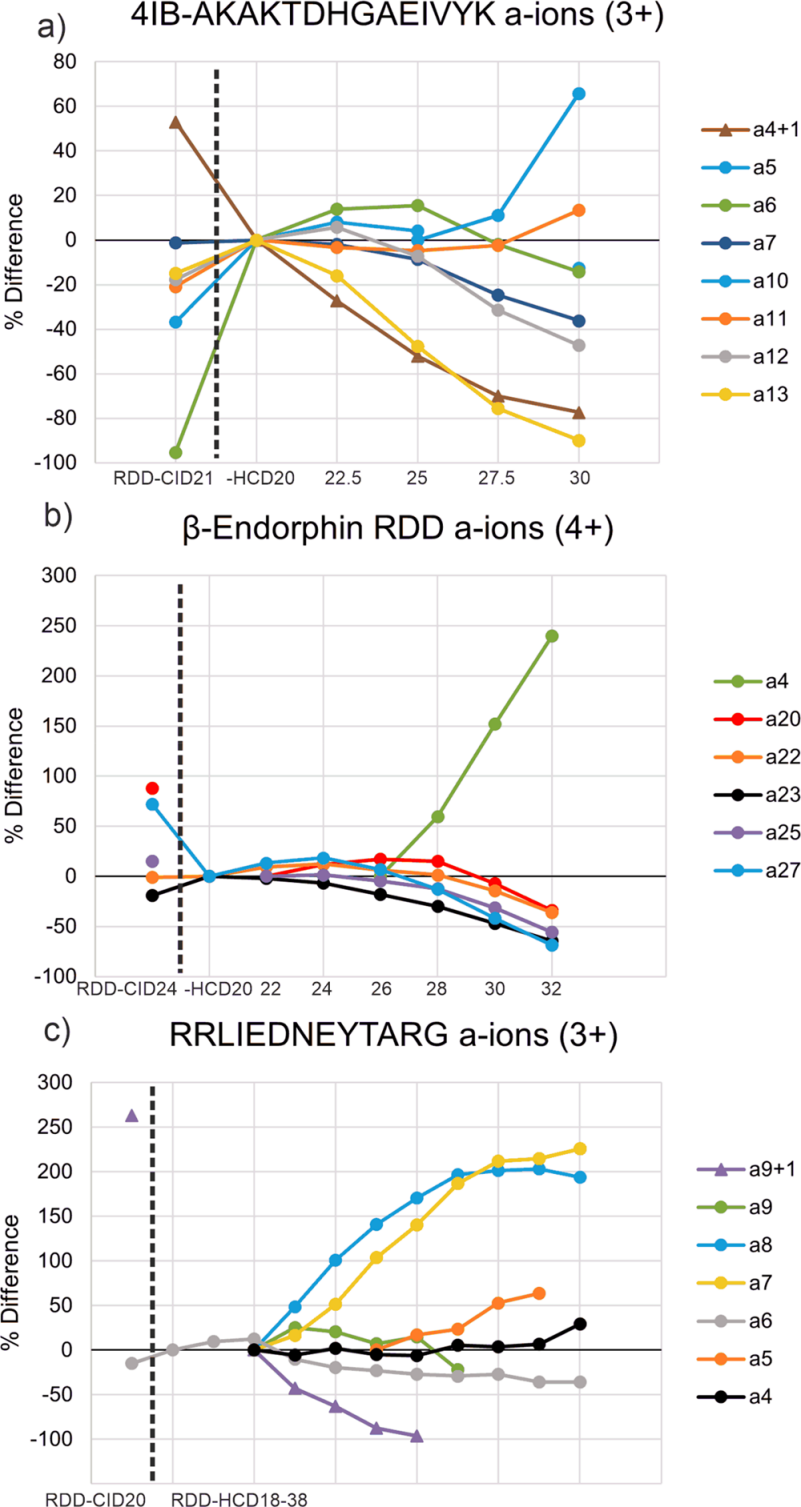
RDD-CID/HCD % change plots for a-ions. (a) 3+ charge state of 4IB-AKAKTDHGAEIVYK, (b) 4+ charge state of β-endorphin, and (c) 3+ charge state of RRLIEDNEYTARG. Radical a+1-species are represented with triangles at each data point, and nonradical a-species are represented with circular points.

RDD favors the production of a-ions at aromatic residues and Ser/Thr due to facile migration to the beta position.^2^ The stability of a-ion products as a function of activation energy is shown in Figure 4. For 4IB-AKAKTDHGAEIVYK in the 3+ charge state, a range of behaviors is observed, including ions that increase or decrease in abundance as a function of HCD energy or between HCD and CID (Figure 4a). Closer inspection reveals that FRAB correlates well with ion length where longer a-ions lose FRAB at higher energies. In contrast, smaller ions tend to increase with higher energy, suggesting that they derive from secondary fragmentation of larger fragments. Notably, the a_4_+1 fragment behaves in completely the opposite fashion, where this shortest fragment decreases in FRAB at higher energies. Importantly, the mechanism that generates this a_n_+1 is known and is specific to sites N-terminal to Ser/Thr residues.^2^ Although typical a-ions are nonradical species, the a+1 is a radical (see Scheme S1c,d). Despite being the shortest fragment (and therefore less prone to additional collisions or secondary fragmentation), the radical nature of the a_4_+1 ion must account for its fragility and reduced FRAB as HCD energy is increased. This interpretation is further confirmed upon consideration of the a-ions generated for 4+ β-endorphin, which includes a typical a_4_ ion (Figure 4b). For β-endorphin, all longer a-ions decrease in FRAB as HCD energy rises, while the a_4_ ion, which does not exist at lower energies, rises dramatically at higher HCD energy. RDD experiments on RRLIEDNEYTARG also confirm these results (Figure 4c). A radical a_9_+1 fragment formed at Thr-10 was observed during RDD-CID and low energy RDD-HCD but decayed in FRAB faster than any of the other a-ions as HCD energy was increased, including the corresponding nonradical a_9_ fragment. Interestingly, a_7_ and a_8_ both increase steadily in fractional abundance with increasing HCD energy. The behavior of these longer fragments with increasing HCD energy could be explained by degradation of the a_9_ and a_9_+1 fragments, which may contribute to production of the a_7_ and a_8_ fragments. A similar pattern is observed for a_4_, a_5_, and a_6_. The a_6_ ion decreases slowly in FRAB as HCD energy is increased, while a_4_ and a_5_ increase moderately in FRAB. This increase in FRAB for a_4_ and a_5_ may be due to degradation of a_6_ to a_4_ and a_5_, in a similar fashion to a_9_.

### Further Consideration of Fragment Size

**Figure 5:**
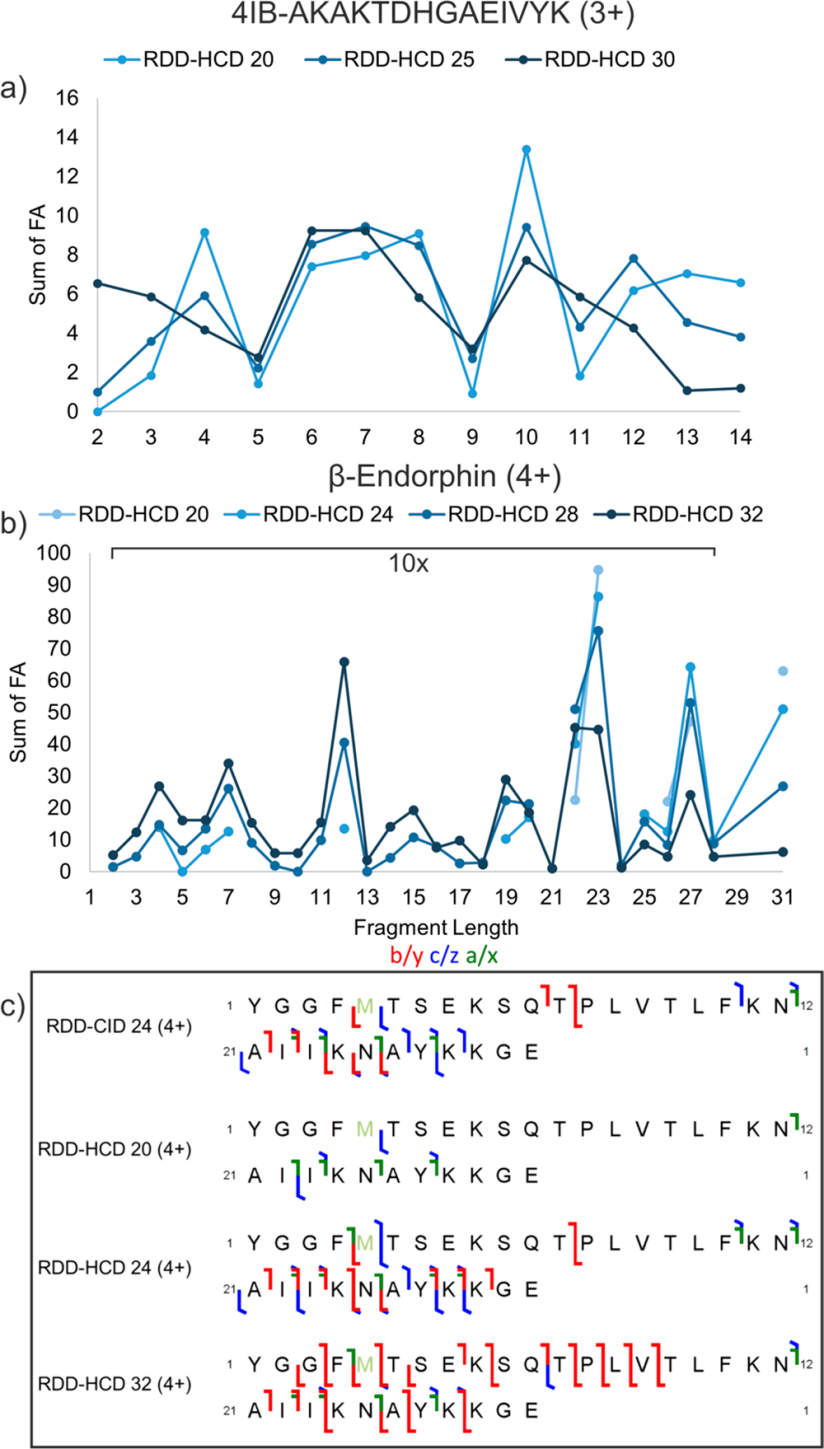
(a) Fractional abundance vs fragment length for 4IB-AKAKTDHGAEIVYK. (b) Fractional abundance vs fragment length for β-endorphin (4+), with the FRAB for fragments 2–28 amino acids in length magnified 10×. (c) Fragment type and sequence coverage for β-endorphin (4+) for RDD-CID24, RDD-HCD20, RDD-HCD24, and RDD-HCD32. b/y fragmentation is shown in red, c/z is shown in blue, and a/x is shown in green.

The decay of larger fragments observed from the neutral losses in Figure 1 shows that there is a potential inverse relationship between fragment size and HCD energy. In order to explore this further, fragment length versus the sum of fractional abundances for all fragments of the same length is plotted for 4IB-AKAKTDHGAEIVYK in Figure 5a at three different HCD energies. At the lowest HCD energy, longer fragments that are 13 or 14 amino acids in length are present in higher FRAB, while these same fragments decrease in FRAB as HCD energy increases. Smaller fragments such as those two, three, five, six, or seven amino acids in length tend to increase in FRAB with increasing HCD energy. This illustrates that higher HCD energy tends to produce smaller ions at the cost of less abundant larger ions. Exceptions to this trend, such as for fragments four amino acids in length, can be rationalized by radical fragility (i.e., the a_4_+1 radical is a major contributor to this data point). For β-endorphin, fragments of length 2–21 are produced in higher FRAB at higher HCD energies, while fragments of length 22–31 are lower in FRAB at higher HCD energies (Figure 5b). It is clear that as HCD energy is increased, smaller fragments begin to dominate the spectrum while larger ones decrease. Examining the types of fragments observed under each experiment also reveals more about what is happening as HCD energy is increased. In Figure 5c, low energy RDD-HCD produces a few c/z and a fragments, and most of these fragments are long. With additional activation, more b/y-, c/z-, and a-ions are made, in addition to shorter fragments. Finally, at even higher energy RDD-HCD, b/y and shorter fragments begin to dominate. These transitions are consistent with diminution of radical fragments and sequential truncation of longer fragments.

## Conclusion

Our results illustrate differences in both the stability of radical versus nonradical fragment ions and differences in the mechanisms leading to fragmentation in CID versus HCD. The resonant excitation used in ion-trap CID does not afford easy access to activation energies exceeding the lowest energy dissociation pathways, meaning that RDD-CID experiments do not vary as a function of activation once the dissociation threshold is achieved. However, the radical peptide fragments produced by RDD are significantly less stable relative to the canonically protonated species (that are also products of RDD) and will easily undergo secondary fragmentation with high energy activation in HCD experiments. The dominant b/y-ions observed at higher HCD energies therefore represent “survivor” ions that have undergone secondary dissociation and have few low-energy dissociation pathways remaining available. Ironically, HCD also appears to enable lower energy activation than is possible with CID, as is most apparent in the second line of Figure 5c. This allows tuning of fragmentation based on HCD energy to obtain spectra with either more or less of a specific fragment type, ranging from radical-dominated products to radical-less products. RDD-HCD therefore appears to be a versatile option to be included in the collection of MS^*n*^ methods.

## Supporting Information Available

Scheme showing selected RDD fragmentation mechanisms, β-endorphin RDD-CID spectra, FRAB plot for 5+ β-endorphin side chain losses, PD-CID-CID on side chain losses from β-endorphin, β-endorphin RDD-CID spectra (PDF)
