# Supplementary material for: RDD-HCD Provides Variable Fragmentation Routes Dictated by Radical Stability
Source: J Am Soc Mass Spectrom. 2023 Feb 14;34(3):452–8. doi: 10.1021/jasms.2c00326 (PMC9982999; doi:10.1021/jasms.2c00326)
Supplement: Supplementary file 1 — js2c00326_si_001.pdf [file js2c00326_si_001.pdf]

Supporting Information For:

RDD-HCD Provides Variable Fragmentation Routes Dictated by Radical Stability

Jacob W. Silzel, and Ryan R. Julian*

Department of Chemistry, University of California, Riverside, California 92521, United States

* Corresponding author: Ryan R. Julian
E-mail: ryan.julian@ucr.edu

Keywords: Fragmentation, photodissociation, radical-directed dissociation, higher-energy collisional dissociation, collision-induced dissociation

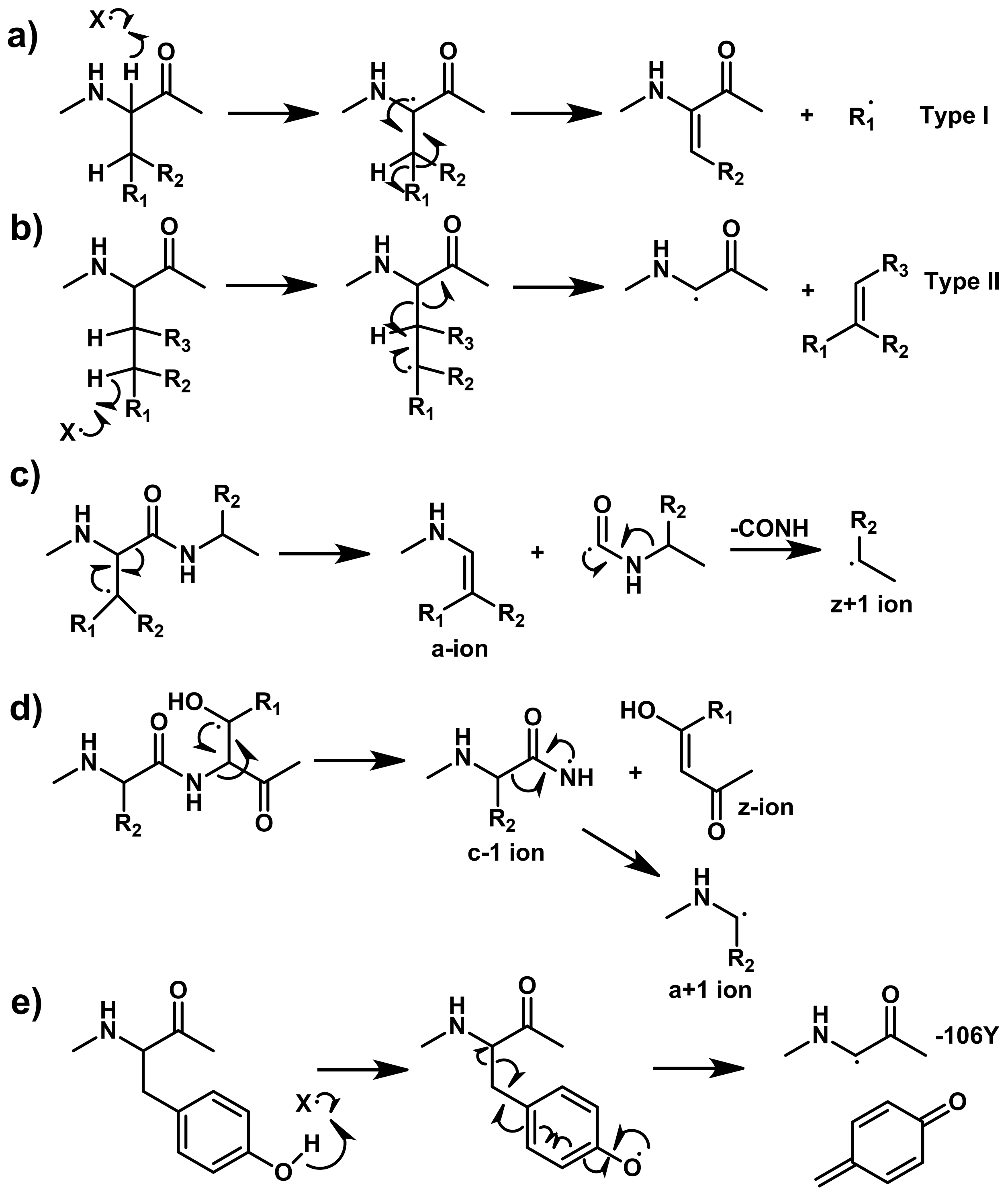

Scheme S1: Selected RDD mechanisms for (a) type I side chain losses, (b) type II side chain losses, (c) a and z+1 ion formation during RDD, (d) a+1 and z ion formation at Ser/Thr during RDD, R_1=H/CH_3, and (e) side chain loss from Tyrosine.

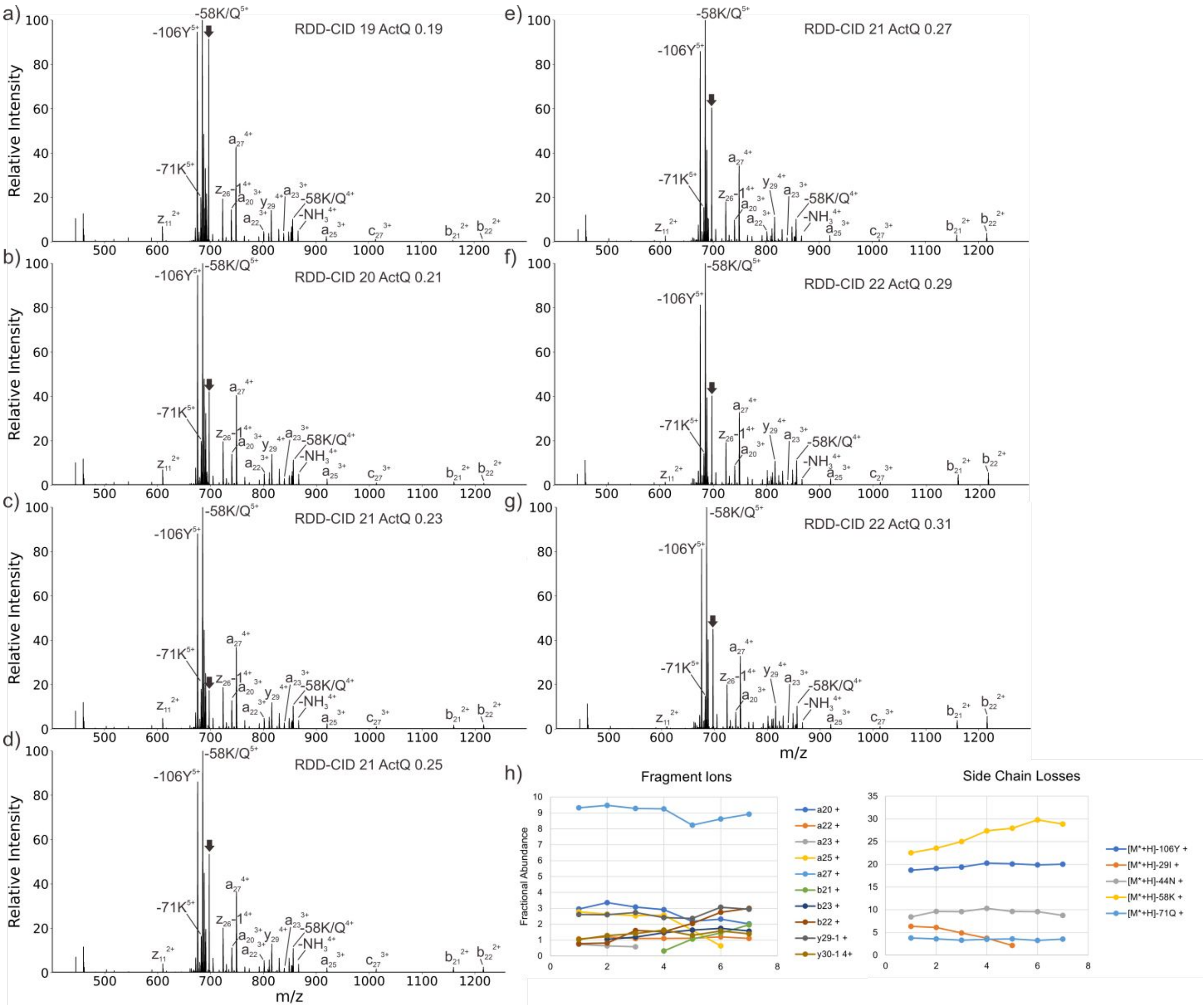

Figure S1: Results of varying RDD-CID energy and Act Q values. Major peaks are labeled and precursor is indicated by the black arrow. a) RDD-CID 19 Act Q 0.19, b) RDD-CID 20 Act Q 0.21, c) RDD-CID 21 Act Q 0.23, d) RDD-CID 21 Act Q 0.25, e) RDD-CID 21 Act Q 0.27, f) RDD-CID 22 Act Q 0.29, g) RDD-CID 22 Act Q 0.31, h) fractional abundance plots versus Act Q values.

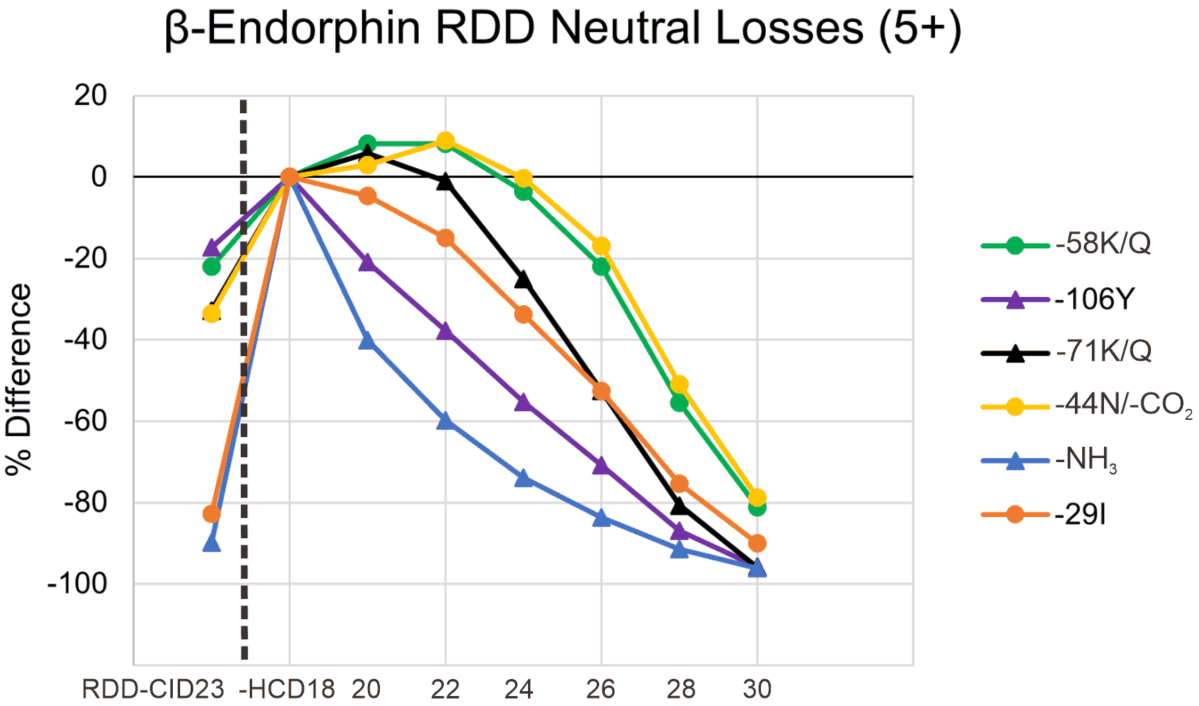

Figure S2: % Difference plot for neutral losses from β-endorphin (5+). Losses in which the radical remains behind on the peptide are denoted by the triangle points, while losses in which the radical has left the peptide with the neutral fragment are denoted with circular points.

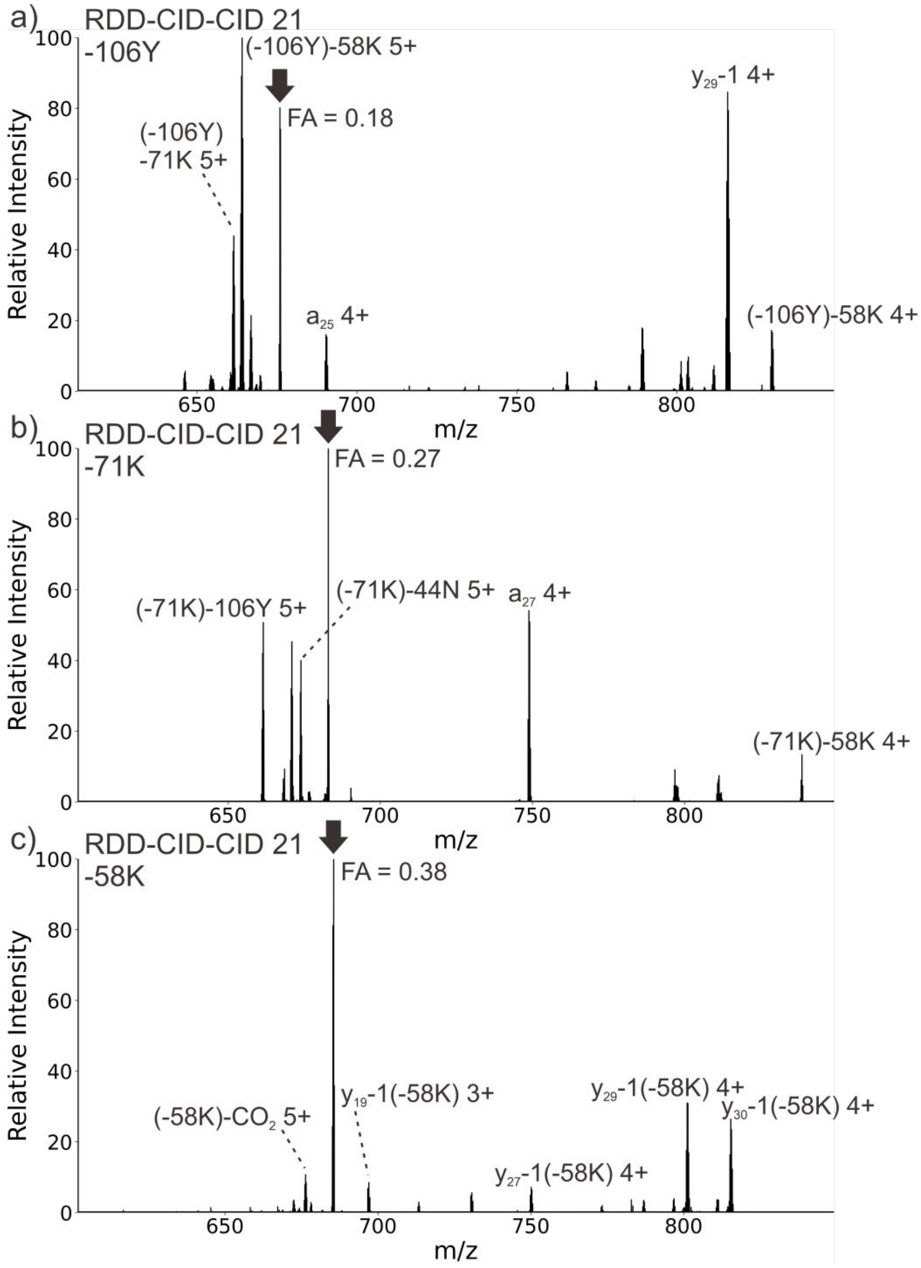

Figure S3: PD-CID-CID (MS4) on a) the RDD-CID -106Y side chain loss from β-endorphin, b) the -71K side chain loss from β-endorphin, and c) the RDD-CID -58K side chain loss from β-endorphin. Fractional abundance (FA) of the remaining precursor is indicated.

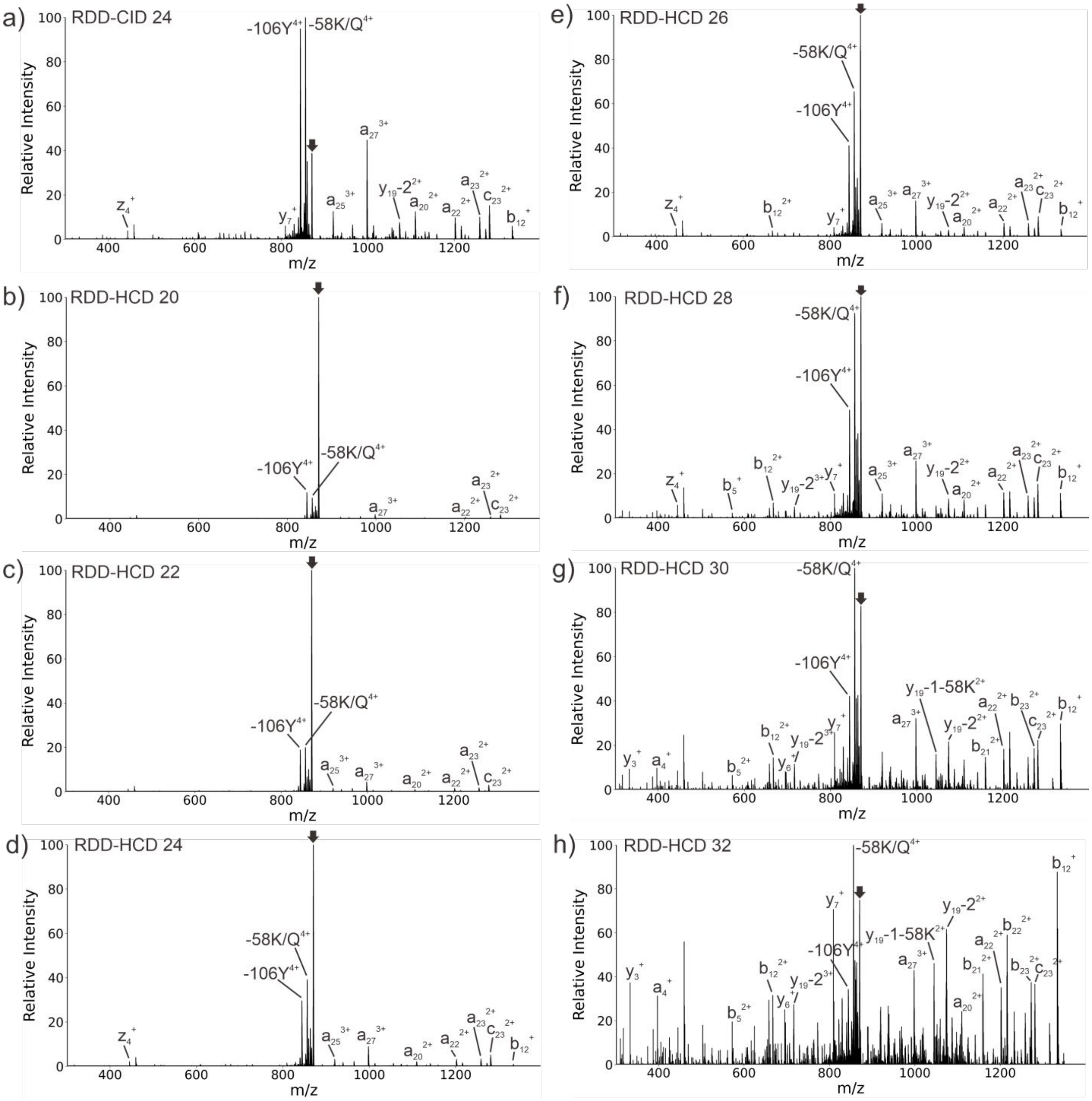

Figure S4: β-endorphin mass spectra for RDD-CID and RDD-HCD at all selected HCD NCE. Major peaks are labeled, black arrow denotes precursor.
